# The effectiveness of a trauma-focused psycho-educational secondary prevention program for children exposed to interparental violence: study protocol for a randomized controlled trial

**DOI:** 10.1186/1745-6215-13-12

**Published:** 2012-02-06

**Authors:** Mathilde M Overbeek, J Clasien de Schipper, Francien Lamers-Winkelman, Carlo Schuengel

**Affiliations:** 1 VU University, Department of Clinical Child and Family Studies, Amsterdam, The Netherlands; 2 EMGO Institute for Health and Care Research, VU University, Amsterdam, The Netherlands

**Keywords:** domestic violence, interparental violence, emotional security hypothesis, posttraumatic stress symptoms, behavioral problems, children, parent-child interaction, community group intervention, RCT, nonspecific factors

## Abstract

**Background:**

Children who witness interparental violence are at a heightened risk for developing psychosocial, behavioral and cognitive problems, as well as posttraumatic stress symptoms. For these children the psycho-educational secondary prevention program 'En nu ik...!' ('It's my turn now!') has been developed. This program includes specific therapeutic factors focused on emotion awareness and expression, increasing feelings of emotional security, teaching specific coping strategies, developing a trauma narrative, improving parent-child interaction and psycho-education. The main study aim is to evaluate the effectiveness of the specific therapeutic factors in the program. A secondary objective is to study mediating and moderating factors.

**Methods/design:**

This study is a prospective multicenter randomized controlled trial across cities in the Netherlands. Participants (N = 140) are referred to the secondary preventive intervention program by police, social work, women shelters and youth (mental health) care. Children, aged 6-12 years, and their parents, who experienced interparental violence are randomly assigned to either the intervention program or the control program. The control program is comparable on nonspecific factors by offering positive attention, positive expectations, recreation, distraction, warmth and empathy of the therapist, and social support among group participants, in ways that are similar to the intervention program. Primary outcome measures are posttraumatic stress symptoms and emotional and behavioral problems of the child. Mediators tested are the ability to differentiate and express emotions, emotional security, coping strategies, feelings of guilt and parent-child interaction. Mental health of the parent, parenting stress, disturbances in parent-child attachment, duration and severity of the domestic violence and demographics are examined for their moderating effect. Data are collected one week before the program starts (T1), and one week (T2) and six months (T3) after finishing the program. Both intention-to-treat and completer analyses will be done.

**Discussion:**

Adverse outcomes after witnessing interparental violence are highly diverse and may be explained by multiple risk factors. An important question for prevention programs is therefore to what extent a specific focus on potential psychotrauma is useful. This trial may point to several directions for optimizing public health response to children's exposure to interparental violence.

**Trial registration:**

Netherlands Trial Register (NTR): NTR3064

## Background

Interparental violence is a considerable worldwide problem; for example in the United States alone 16% of all children (2-17 years of age) witness partner assault some time in their childhood [1]. The latest estimates within the Netherlands are that 12% of all adolescents have witnessed interparental violence in their lives [2]. The consequences of witnessing interparental violence are severe; meta-analyses show that children exposed to interparental violence experience emotional, behavioral and cognitive problems [3,4]. Children exposed to interparental violence may show short-term maladjustment as well as develop long-term mental health problems. For example, adults who witnessed interparental violence as children are two to four times more likely to report problems with alcoholism, drug use and depression [5]. Despite potential severe and lifelong consequences of witnessing interparental violence, few carefully designed interventions for children exposed to interparental violence have been developed, and even fewer of these programs have been thoroughly tested [6]. Questions remain therefore about the kind of interventions that may prevent or limit adverse consequences for children exposed to interparental violence.

According to Cummings and Davies' (2010) emotional security hypothesis, exposure to destructive interparental conflict increases children's vulnerability to psychological problems by undermining their confidence in the interparental relationship and the security they find in the family. Direct effects derive from experiencing the conflicts themselves; indirect effects derive from experiencing deteriorated parenting and parent-child interaction due to the effects of conflict on the parents. Preserving feelings of emotional security is a salient psychological goal for children, governed by emotional and behavioral regulation and cognitive representations. These response processes may to some degree help to defend their physical and psychological safety, but may have long-term costs or break down in the end. Children's emotional and behavioral regulation in the face of interparental violence was longitudinally associated with children's externalizing and internalizing problems [7].

Children may embark on a developmental pathway towards psychopathology, even after interparental conflict has stopped. These effects are likely to depend on specific family, parent or child factors. The degree of children's adjustment problems may depend on the duration and severity of the domestic violence [3,8], parenting and psychological functioning of the parent [9]. Research is inconsistent regarding the impact of demographic variables, such as age, gender and ethnicity on children's adjustment after witnessing interparental violence [10,11].

Based on the emotional security perspective, there is a need for preventive interventions that restore security in the family system(s) that children find themselves in, and that support the parents and improve parent-child interaction [7]. Such programs may diminish the long-term consequences of interparental violence.

Trauma theory [12] focuses on how experiences that elicit overwhelming fear alter affective functioning. Usually, distress after a frightening experience is transient. If distress persists, a posttraumatic stress disorder (PTSD) may develop. Symptoms of a posttraumatic stress disorder in adults as well as in children include 1) re-experiencing the trauma, as flashbacks or nightmares; 2) persistent avoidance of stimuli associated with the trauma and emotional numbing; and 3) persistent hyperarousal, evidenced by sleeping and concentration problems, irritability and hypervigilance [13]. Therapeutic intervention therefore focuses on readjusting affective responses to trauma-related thoughts and memories, as well as reminders. Techniques are therefore not only based on trauma theory, but also on social cognitive theory [14], for example within Trauma Focused Cognitive Behavioral Therapy (TF-CBT) for children diagnosed with PTSD. Components used in TF-CBT for children are psycho-education, relaxation skills, affective modulation skills (e.g. identification of feelings), cognitive coping and processing, enhancing future safety and development, and trauma narrative. Affective functioning in children is closely aligned with relationships with parents, and therefore TF-CBT includes psycho-education of parents, parenting skills training and conjoint child-parent-sessions, in which the child shares the trauma narrative and other family issues are addressed [15]. The effectiveness of TF-CBT is well established for victims of sexual abuse [16]. For children exposed to interparental violence, Cohen and her colleagues [17] compared in a randomized controlled trial a brief (8 weeks) individual Trauma-Focused Cognitive Behavioral Therapy to Child Centered Therapy (CCT) and found that children who completed TF-CBT showed improvement in PTSD symptoms and anxiety symptoms relative to children who completed CCT.

Further support for the role that parent-child relationships may have for risk, resilience and recovery in the case of exposure to interparental conflict comes from attachment theory [18]. Therapy with child and parent includes components such as assisting the parent with accurate interpretation of the child's feelings and actions, providing emotional support to parent and child, and developing a joint parent-child narrative about the trauma. The effectiveness of attachment theory in intervention for preschoolers exposed to interparental violence, has been studied by Lieberman and her colleagues in a randomized controlled trial. They found that a 50-week child parent psychotherapy program reduced traumatic stress symptoms and improved behavior compared to case management with individual psychotherapy.

One problem with therapy for children exposed to interparental violence is that access to such therapy is contingent on linking behavioral dysfunction with exposure to the violence. A child has to be seen as functioning problematically and the professional has to be aware of the role of the violence in behavioral dysfunction before a child will be referred to such therapy. Also, children and their families differ in their tendency to seek treatment after exposure to traumatic events [19]. Therefore, a secondary prevention program, 'Kids' Club', was developed in the United States [20] that is offered to families with children who seek help or refuge after interparental violence, or who have been involved with social services or police because of family violence. Children do not have to exhibit problematic functioning for participation. The program was mainly based on insights from trauma theory, later including parallel parent sessions, given recognition of the importance of parents for resilience and recovery. Effectiveness of 'Kids' Club' has been studied in a quasi-experimental study by Graham-Bermann and her colleagues [21]. They found that this community based group intervention of 10 sessions was effective in reducing externalizing and internalizing behavior problems, compared to a waitlist group. Furthermore, children in the condition with parallel parent and child sessions, showed greater reduction in behavioral problems compared to children following a similar intervention but with only child sessions and no parent sessions [21]. These results support the inclusion of parents in interventions for children exposed to interparental violence. The original child sessions of 'Kids' Club' have been used as a basis for the development of a secondary prevention program in the Netherlands. Independent from 'Kids' Club', the Dutch program was further developed by including nine parallel parent sessions. This program is called 'En nu ik...!' ('It's my turn now!'). Lamers-Winkelman showed in a study with a quasi-experimental design that children, after participation in the program 'En nu ik...!', exhibited less internalizing and externalizing behavioral problems and less posttraumatic stress symptoms [22].

To our knowledge, no randomized controlled trial on the effectiveness of this secondary prevention program has been carried out. The only two randomized controlled trials studying therapeutic interventions for children exposed to interparental violence [17,18], suggest that a focus on trauma as well as on the parent-child relationship in therapy is important to reduce adjustment problems in children. The question is whether this may be true for secondary prevention programs as well, because the population for preventive intervention is likely to be more diverse in symptomatology at referral than the population for therapeutic intervention. Another question regards the effect of specific factors aimed at the traumatic experience compared to nonspecific factors. Previous trials compared different specific factors or different combinations of specific and nonspecific factors. As a result, it is unknown whether for children often exposed to multiple risk factors besides interparental violence [7], a focus on the traumatic experience can be shown to have added value.

To study the effectiveness of the secondary prevention program 'En nu ik...!' and to assess the need for a specific focus on trauma in interventions, a randomized controlled design is used within this study. Parents and children are randomly assigned to either the existing secondary prevention program or an alternative control program. This alternative program has been developed for this study and has the same structure as the secondary prevention program, but has been stripped of any specific factors, does not include a trauma-focus and is solely based on nonspecific factors in interventions, such as positive attention, recreation, distraction, warmth and empathy of the therapist, and social support among group participants [23].

In all aforementioned studies about the effectiveness of interventions for children exposed to interparental violence, only questionnaires were used to gather information, and in the study of Lamers-Winkelman (2003) [22] and Cohen et al. (2011) [17] no follow-up was conducted. In this study, observational measures as well as questionnaires for parent, child and teacher, are used to prevent a reporting-bias, and follow-up assessments are included to assess long-term consequences of participation.

In addition to the study of program effectiveness, evidence for mechanisms of change and predictors of effectiveness may further support the intervention design. Graham-Bermann and her colleagues (2011) pioneered the study of specific mediators and moderators, focusing on parenting and mental health of the mother as mediators and degree of exposure to family violence and demographic variables as moderators [10]. The current study will focus on parent-child interaction, emotional security and taught skills (coping strategies, feelings of guilt, the ability to differentiate and express emotions) as mediators and mental health of the parent, parenting stress, disturbances in parent-child attachment, duration and severity of the domestic violence and demographics as moderating variables.

### Trial objective

In order to justify broad implementation of a protocol led psycho-educational secondary preventive intervention program for children exposed to interparental violence in the Netherlands, empirical evidence is needed to show that children benefit from participation in this program. The present study will add to this evidence with a randomized controlled trial (RCT). Effectiveness is measured in terms of less posttraumatic stress symptoms, less internalizing and less externalizing behavioral problems. Because of the design of this study in which children in a trauma-focused intervention program are compared to children in a control program with only nonspecific factors, this study will provide insight in the need for trauma-focus in intervention. This knowledge can be used to improve care for children exposed to interparental violence. Potential mediating and moderating variables are investigated in order to evaluate underlying mechanisms of change and to identify predictors of effectiveness.

## Methods

### Study design

The study is designed as a multicenter trial with concealed random allocation to the experimental arm which will receive the secondary prevention program 'En nu ik...!' ('It's my turn now!') and the control arm which will receive a program similar in form, but containing only nonspecific elements, called 'Jij hoort erbij' ('You belong'). The multicenter design was chosen to improve generalizability of the study results. Eight organizations in seven cities in urban and rural regions of the Netherlands have agreed to participate in this research.

### Randomization

An independent researcher will make the allocation schedule with a computerized random number generator. The random allocation list will be generated in blocks of no less than two, depending on the number of participating groups per time period. The numbers on the list will be paired with the groups in order of the date on which the groups start. The condition of the group will be disclosed to the participating organizations and to the researchers two weeks before the start of the program. Potential contamination of the intervention and control group will be avoided by restricting recruitment to one child per family.

### Study population

140 children (6-12 years of age) and their caregiving custodial parents who experienced interparental violence will participate in this study.

### Inclusion and exclusion criteria

Children and their parents who experienced interparental violence and who are willing to take part in a secondary prevention program are eligible for participation in the study. Inclusion criteria to participate in the program and the study are: having experienced interparental violence, the violence has stopped at the time parent and child start with the program, and parent(s) has/have given informed consent to take part in the study. Exclusion criteria are: child/parent has such intellectual, psychiatric or behavioral problems that the behavior will impede functioning within the group and/or will create unsafety in the group for all participants.

### Procedure

In the Netherlands, parents and children who experience interparental violence are referred to a social service specialized in assisting families exposed to interparental violence in their neighbourhood (e.g. ASHG: Support Center for Domestic Violence, Women Shelters or Mental Health Care organizations) by police, other social services, youth care workers and health care professionals. These organizations offer, among other services, the intervention program. During the intake for the intervention program, all parents and children are informed that the organization offers two programs for children exposed to interparental violence in the study-period (intervention and control program) and they will be informed in which program they will participate as soon as possible. Information is provided about the study and every parent-child-dyad eligible for participation in the program is asked to participate in the study. If parent and child agree to participate in the study, they are asked to sign a consent form. If both parents have custody over the child, by Dutch law the other custodial parent needs to be informed about participation of his/her child in the study. The caregiving parent is asked to inform the other parent by means of an information brochure and letter from the researchers. In this letter the other parent is asked to give written consent for participation of the child in the study. Due to the law on privacy, the researchers are not allowed to contact the other parent directly. If no response of the other parent is received, and the caregiving parent has given written consent for participation, the child will be included in the study. This procedure has been established with the approval of the Medical Ethics Committee (METc VUmc 2009/99/NL26649.029.09), because of the specific problems in the family situation of the population participating in the study. If parent and child choose not to participate in the study, they can still enroll in the program.

**Figure 1:**
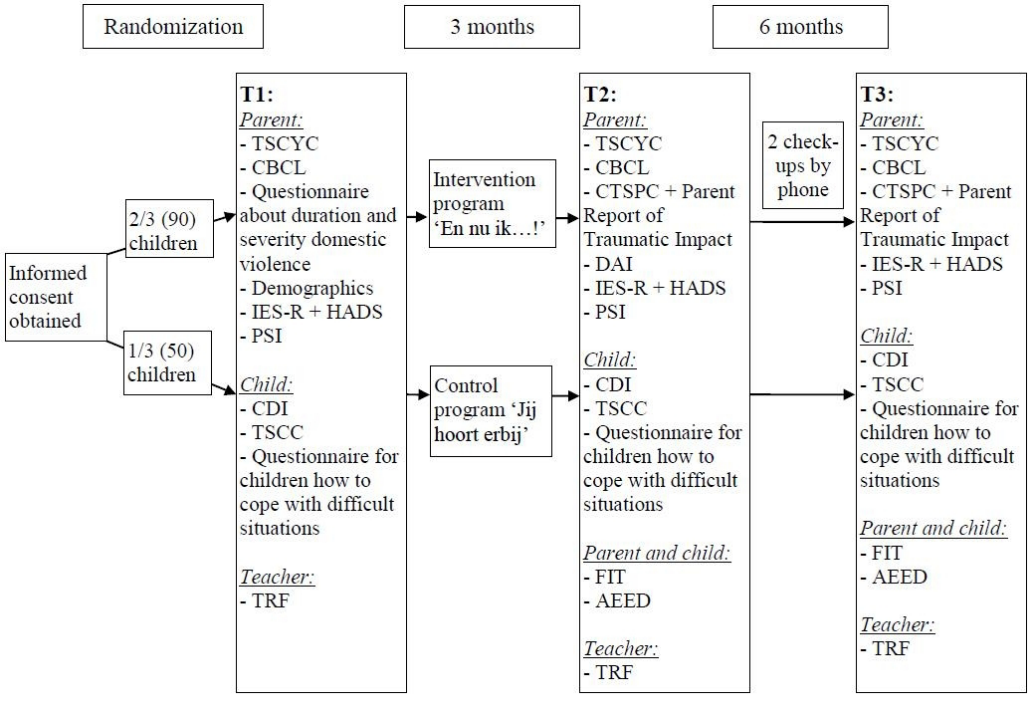
**Research procedure**. After informed consent is obtained, 2/3 of all parent-child dyads will be randomized into the experimental arm and 1/3 into the control arm. Parents and children are asked to fill out questionnaires (parent: TSCYC, CBCL, Questionnaire about duration and severity of domestic violence, demographic questionnaire, IES-R, HADS, PSI; child: CDI, TSCC, Questionnaire for children how to cope with difficult situations) at the first assessment (T1). A week later the program starts for all parents and children. One week (T2) and six months (T3) after the end of the program parents and children who take part in the study are again invited to fill out questionnaires and participate in two observation tasks (FIT and AEED). To check up on parents in the period between T2 and T3, parents are contacted by phone two times. At all assessments the teacher is also sent a questionnaire.

Each group is randomly assigned to either the intervention or the control arm. Parents and children are given the details about the program into which they are randomized. Parents and children who participate in the study are invited to the setting (e.g. community centre, mental health clinic) at which the program (intervention or control) takes place one week before the program starts. They are asked to fill out questionnaires (T1). A week later the program starts for all parents and children. One week (T2) and six months (T3) after the end of the program parents and children who take part in the study are again invited to fill out questionnaires and participate in two observation tasks. If parents or children have trouble filling out the questionnaires, a trained masters student or an interpreter will assist. To check up on parents in the period between T2 and T3, parents are contacted by phone two times. Figure 1 shows the different stages of the research procedure and gives an overview of the instruments used at each assessment.

Parents receive €15,- after completing the first assessment, €25,- after participating in the program and completing the second assessment and €40,- after completing the follow up assessment. Children receive a gift after each assessment.

### Blinding

Parents, children, social workers as well as the researchers are blind to group allocation until two weeks before the start of the program. The condition is not disclosed earlier to avoid a bias in the intake procedure because of prior knowledge about the condition. Parents and children agree to participate in the study before randomization and without knowing in which arm they will be enrolled. Both programs are presented as useful programs by discussing the specific and/or nonspecific factors that can be of benefit to children and parents exposed to interparental violence. Every ID-number assigned to a parent-child-dyad is independent of the condition. The researchers coding the observation tasks and analyzing the data will be blind to the group condition of parents and children, as well as assessment (T1, T2, T3).

### Interventions

#### Intervention program 'En nu ik...!' ('It's my turn now!')

The child sessions of the program 'En nu ik...!' are based on the psycho-educational secondary preventive intervention program 'Kids' Club' [20]. Changes in the original manual, with respect to topics and methods have been made. 'En nu ik...!' was further developed by including nine parallel group sessions for parents. Theoretical conceptualizations used in the development of this program are trauma theory [12] and attachment theory [18]. Child sessions of 'Kids' Club' have been adapted for use in the Netherlands by a consortium formed by the Women Shelter in Amsterdam (Blijf Groep), the Centre of Youth Care in Amsterdam (BJAA/SO&T), social work and mental health care services in Amsterdam (the Netherlands). The program consists of nine group sessions of 90 minutes each for children and nine parallel group sessions for their caregiving custodial parent. Each group has a maximum of eight children or parents. Usually a social worker together with a mental health care professional act as therapists. Therapists are trained in giving this standardized program and follow a protocol for every session. Therapists receive supervision during the program.

##### Children's sessions

The program 'En nu ik...!' has three main intervention goals for children:

 1) to (make a start with) process(ing) the interparental violence experiences

 2) to learn how to differentiate and express emotions

 3) to learn how to cope with feelings and problems in a different (non-violent) way

 All sessions follow a predictable structure. Every week the children start in a circle and hold hands to greet each other. Then they are asked how they feel today and a short story about different topics related to interparental violence and emotion recognition is read and talked about. After that the children have a break and drink and eat something before they start with the activity of the week. Every activity is related to the topic covered that week and varies per session. The session is finished with a gross motor game to relieve tension after a possible strenuous session. Children and parents are reunited and encouraged to share what they have done during the session. Focal topics are: session 1) getting to know each other and recognizing emotions; 2) emotions; 3) sadness or happiness and a safe place; 4) anger; 5) conflicts and loyalty; 6) violence, conflicts and contact with the other parent; 7) secrets and safety; 8) the future, and 9) saying goodbye and evaluation.

##### Parents' sessions

Goals for the parents' sessions are to learn how to become more sensitive in supporting their children who deal with difficult experiences and emotions, and to take the perspective of the child who witnessed interparental violence. Although the parent sessions are not aiming to help parents process their own experiences, sharing of experiences is not discouraged either and a supportive group atmosphere is created. Focal topics of the sessions are: 1) getting to know each other; emotions; violence; 2) parenting: the parenting role versus the role of the child; 3) a safe place, offering safety, making compliments; 4) coping with feelings of anger and sadness; 5) coping with feelings of guilt, shame and loyalty; 6) safety, conflicts and contact with the other parent; 7) social contacts and social network; 8) unspecified theme, depending on the needs of the participants, and 9) saying goodbye and evaluation.

#### Control program 'Jij hoort erbij' ('You belong')

For this study, an alternative control program was developed, based on a content analysis of nonspecific factors within the intervention program [23]. Basic elements of interventions, including positive attention from a therapist, positive expectations and hope, distraction and social support and recognition among group participants were deemed present in the intervention program, and were therefore built into the design of the control program as well; no specific attention is paid to the interparental violence. The reasons for the development of a control program were 1) to evaluate the need for specific factors in interventions, 2) to be able to monitor families in both arms equally well over time, and 3) to prevent higher drop-out rates in the control arm.

The control program follows the same structure as the intervention program and also consists of nine sessions of 90 minutes each for children and nine parallel group sessions for their caregiving custodial parent. The maximum group size is eight participants. Therapists are a clinical child psychologist/pedagogic specialist and a pedagogic specialist in training, for both parents and children. Therapists are trained in giving this standardized program and receive supervision during the program.

##### Children's sessions

In the children's sessions of the control program, a predictable and structured positive atmosphere is created. The control program follows a comparable structure as the intervention program: the children start in a circle holding hands, then they participate every week in a different fun activity. After that, children have a break and drink and eat something, listen to and discuss a short story about a topic unrelated to interparental violence or emotion recognition, and end with a gross motor game. At the end of the session, children are reunited with their parents and share their activities. The themes of the sessions are: 1) getting to know each other; 2) playing games; 3) healthy food and painting dinner plates; 4) decorating a name plaque; 5) relaxing: watching a movie/playing video games; 6) painting a club shirt; 7) scouting expedition; 8) making a goodbye movie, and 9) saying goodbye and evaluation.

##### Parents' sessions

To be able to relate changes in outcome measures to the content of the program, also nine parents' sessions have been developed for the control program. As in the children's sessions, the focus in the parents' sessions is on creating a positive atmosphere and having a good and relaxing time together. Topics of the sessions are: 1) getting to know each other; 2) fun things to do with your child(ren); 3) healthy food; 4) do-it-yourself; 5) books to read together with your child(ren); 6) how to keep a budget; 7) creative activities; 8) bingo, and 9) saying goodbye and evaluation.

### Measures

In this study primary outcome measures, mediating variables, moderating variables and control variables can be distinguished. The instruments include several questionnaires for both parent and child, and one for the teacher, two observational measures with parent and child, and an interview with the parent.

#### Primary outcome measures

The main research question of this study is whether children who experienced interparental violence benefit from participating in the intervention program in terms of less posttraumatic stress symptoms, less internalizing behavioral problems and less externalizing behavioral problems in comparison with children participating in the control program.

##### Posttraumatic stress symptoms of the child

To assess posttraumatic stress symptoms of children, parents are administered the Dutch translation of the **Trauma Symptom Checklist for Young Children (TSCYC) **[24]. This questionnaire consists of 90 items; parents rate the behavior and emotions of their child in the past month on a 4-point Likert scale, ranging from 'not at all' (1) to 'very often' (4). The TSCYC consists of eleven scales: two scales to assess the validity of the parent's answers (response level and atypical response), eight clinical scales (anxiety, depression, anger/aggression, PTSS-intrusion, PTSS-avoidance, PTSS-arousal, dissociation and sexual concerns) and a total PTSS score. The clinical scales of the TSCYC showed good reliability within a sample of maltreated children in the United States (Cronbach's α = .81-.91) [25] and in the Netherlands (Cronbach's α = .79-.91) [26].

To assess self-reported posttraumatic stress symptoms children are administered the Dutch translation of the **Trauma Symptom Checklist for Children (TSCC) **[27]. This questionnaire consists of 54 items, clustering in eight scales: two validity scales (underresponse, hyperresponse) and six clinical scales (anxiety, depression, anger, posttraumatic stress, dissociation, sexual concerns). The response categories are the same as in the TSCYC and reliability was high, with Cronbach alpha's ranging from .78 to .86 in a sample of sexually abused children [27]. In a sample of maltreated children in the United States TSCC showed discriminant and convergent validity with the TSCYC [28], and in the Netherlands TSCC showed convergent and criterium validity with other behavioral questionnaires (CBCL, TRF, YSR, CDI) [29].

##### Internalizing and externalizing behavioral problems of the child

Parents and teachers report about internalizing and externalizing behavioral problems of children using the **Child Behaviour Checklist (CBCL) **and the **Teacher Report Form (TRF) **[30]. Both parents and teachers are asked to fill out this questionnaire to collect information about the behavior of the child in two settings. The CBCL/TRF is often used in research on the effectiveness of programs for traumatized children [17,21] and has proven to be valid and reliable in research with normative and clinical populations. Cronbach alpha's for the broadband and total scales in a Dutch sample ranged from .78 to .93 for the CBCL [31] and from .86 to .96 for the TRF [32]. The CBCL consists of 113 items with which the parent rates the behavior of the child on a 3-point scale, consisting of 'not true'(0), 'sometimes true'(1) and 'very/often true' (2). The broadband scale 'internalizing problems' consists of anxiety/depression, withdrawal and somatic complaints subscales; the broadband scale 'externalizing problems' consists of aggression and delinquency syndrome subscales. Thought problems, social problems and attention problems make up the other subscales. All items can be summed to compute a 'total problems' score [30].

Children are administered the **Child Depression Inventory (CDI) **[33], consisting of 27 items to assess depression symptoms. Per item the child is asked to choose one of three sentences that fits best with his/her feelings and thoughts in the past two weeks. The child's answers are calculated into a total score (ranging from 0 to 54). The internal consistency in a Dutch sample was high (α = .79), just as the test-retest reliability (r = .79). The CDI has high criterion validity and scores on the CDI correlate highly with scores on other measures for depression [34].

#### Measures of mediating variables

##### Parent-child interaction

To observe the interaction between parent and child, the **Family Interaction Task (FIT**) [35] is used. This observation measure consists of four tasks (guessing game, an enticing marble game (labyrinth), planning a birthday party and a difficult puzzle) and measures affective, behavioral and verbal interaction. The tasks are scored on three scales for the parent (positive responsiveness; quality of assistance; anger and hostility), three scales for the child (expressions of positive affect; persistence and diligence; anger, defiance and frustration) and three dyadic scales (collaboration and teamwork; dyadic negative affect; dyadic positive affect). Every scale ranges from 1 to 5; 1 represents a low score and 5 a high score on a specific dimension. Scores are assigned based on the whole session. Interrater reliability in previous studies ranged from .63 to .73 with children in their middle childhood in the United States [35] and from .87 to .94 with adolescents in the Netherlands [36].

To assess whether parent and child can construct a constructive narrative about emotions, a topic to which a lot of attention is paid in the intervention program, parent and child are asked to participate in the **Autobiographical Emotional Events Dialogue (AEED) **[37]. Within this task parent and child are asked to describe together four situations in which the child felt four different emotions (happy, sad, angry and scared). The narratives are scored on seven scales for the parent and seven parallel scales for the child (shift of focus; boundary dissolution; acceptance and tolerance; cooperation, involvement and reciprocity; hostility; resolution/closure of negative feelings; elaboration and structuring), and two overarching scales (adequacy of the story; coherence). Every scale ranges from 1 to 9 and the higher the score, the higher the level of the specific behavior. Parent and child can be classified as 1) emotionally matched, 2) emotionally unmatched: excessive, 3) emotionally unmatched: flat, and 4) emotionally unmatched: inconsistent. Interrater reliability in previous research ranged from .87 to .95 [38].

##### Treatment components in the children's sessions of the intervention program

##### Emotional security

The main goal of the children's sessions of the intervention program is for children to learn how to cope with their experiences and feel safe again. The **Security in the Family System scales (SIFS) **is a questionnaire especially developed to assess feelings of security within the family subsystem. To assess whether children feel safer after completing the program, they are administered the scale 'secure' of the SIFS. The internal consistency of this scale was .85 and the test-retest reliability was .82 in previous studies [39].

##### Coping strategies

In the children's sessions of the intervention program children are taught to seek support from significant others in times of need. Another part of the program is to play a game with the use of gross motor skills to release tension, after discussing possibly difficult subjects. Within the program children also learn to rephrase destructive thoughts such as feeling responsible for the occurrence of the interparental violence. Three of the five scales of the **How I Coped Under Pressure Scale (HICUPS) **[40] ask about these three strategies (seeking support, distraction and rephrasing thoughts). In another study the internal consistency of the Dutch translation of these scales was .77 for 'Distraction', .86 for 'Positive cognitive restructuring' and .88 for 'Seeking support' [41].

##### Feelings of guilt

Many children who experienced interparental violence feel guilty about what happened [42]. In the intervention program children are taught that the interparental violence was not their fault. To assess whether children internalize this message and feel less guilty after completing the program, they are administered the scale 'Self-blame' of the Dutch questionnaire **Cognitive Emotion Regulation Questionnaire (CERQ-k)**. Internal consistency of this scale was .79 in previous research [43].

##### Emotions

In every session children learn about different emotions, how to recognize these emotions and how to express them in a safe and constructive way. Children are asked to fill out the scale 'Differentiating Emotions' of the **Emotion Awareness Questionnaire (EAQ) **[44] to assess their ability to differentiate emotions. The internal consistency of this scale was .67 in a Dutch sample in a previous study [44]. To assess progress in the ability to express emotions, parents and children also participate in the observation task AEED [37] (for a detailed description see under 'parent-child interaction').

To assess possible effective elements of the intervention program all items of scales mentioned above, are combined in one questionnaire: 'Questionnaire for children about how to cope with difficult situations'. To assess whether children generalize learned coping strategies to different situations, they are asked to fill out the questions about coping strategies for two different situations: a situation in which they had an argument with one of their parents and a situation in which they had an argument with a friend, both in the past two weeks. To assess feelings of guilt in both situations the scale 'Self-blame' of the Cognitive Emotion Regulation Questionnaire is also administered for both situations.

#### Measures of moderating variables

##### Mental health of the parent

Posttraumatic stress symptoms of the parent are assessed with the Dutch version (translated by Olff, 2006) of the **Impact of Events Scale-Revised (IES-R) **[45]. This questionnaire consists of 22 items divided in three scales: intrusion, avoidance and hyperarousal. Parents rate their own behavior and thoughts of the past seven days on a 5-point Likert scale, ranging from 'not at all' (0) to 'very much'(4). Test-retest reliability (.76) and internal consistency (.94) were high in other research [46].

To assess anxiety and depression symptoms of the parent, the Dutch version of the **Hospital Anxiety and Depression Scale (HADS) **[47] is used. This screening instrument consists of seven items concerning depression and seven items concerning anxiety. Respondents are asked to answer on a 4 point scale and the total score of both subscales is interpreted. In other studies within the Netherlands internal consistency ranged from .82 to .90 for the 'Total score', from .80 to .84 for the subscale 'Anxiety', and from .71 to .86 for the subscale 'Depression'. The test-retest reliability (after three weeks) for the 'Total score' was .91, for the subscale 'Anxiety' was .89 and for the subscale 'Depression' was .86 [48].

##### Parenting stress

Parents who experience a lot of parenting stress may have less psychological resources left to benefit from the intervention. The overall parenting stress is measured with the short version of the **Parenting Stress Index (PSI) **[49], translated into Dutch [50]. The questionnaire consists of 25 items and covers child related stress as well as stress related to the parental role. The parent is asked to answer on a 6 point scale, ranging from 'totally disagree' (1) to 'totally agree' (6). The PSI can be used with parents of children aged 3-12 years. Internal consistency in previous studies ranged from .92 to .95 and validity seems acceptable [50].

##### Disturbances of attachment

To assess disturbances in the attachment relationship between parent and child after experiencing interparental violence, the parent is interviewed with the **Disturbances of Attachment Interview (DAI) **[51]. This interview is conducted by phone after T2. This semi-structured interview consists of twelve questions assessing symptoms of inhibited disturbances of attachment (5 items), disinhibited disturbances of attachment (3 items) and secure base distortions (4 items). A question is scored with 0 if the symptom is not present, 1 if the symptom is partly present, and 2 if the symptom is definitely present. Interrater reliability ranged from .71 (disinhibited) to .86 (inhibited) and internal consistency ranged from .67 (disinhibited) to .80 (inhibited) in a study with a sample of Dutch foster children [52]. Internal consistency of the secure base distortions taken together was .75 [53].

##### Duration and severity of the domestic violence

To determine what violent acts have occurred within the interparental relationship, parents are asked to fill out three scales ('Negotiation' (α = .86), Psychological aggression' (α = .79) and 'Physical assault' (α = .86)) of the **Revised Conflict Tactics Scales (CTS2) **[54]. A few questions about the duration of the interparental violence were added.

To assess whether children experienced other forms of child abuse or traumatic events, besides witnessing interparental violence, the **Conflict Tactics Scales Parent-Child (CTSPC) **(scales 'Overall physical assault towards the child' (α = .55), 'Psychological Aggression towards the child' (α = .60), 'Nonviolent discipline (α = .70), 'Neglect' (α = .22)) [55,56] and the **Parent Report of Traumatic Impact **[57] are administered to the parent.

To assess child abuse and neglect in the own childhood of the parent the **Adverse Childhood Experiences (ACE)-questionnaire **[58] is administered.

##### Demographic variables

A demographic questionnaire is used to collect participant's demographic information. This questionnaire consists of 14 questions concerning ethnicity, living situation, education, social economic status and age and gender of the child.

All questionnaires for parents and children are combined in an attractive booklet. All instruments will be administered at all three assessment moments, with the exception of the demographic variables (only at T1) and part of the questionnaire about duration and severity of domestic violence (CTS2 and ACE only at T1), the interview about disturbed attachment behavior (only at T2) and the observation tasks FIT and AEED (not at T1).

Figure 1 provides an overview of the instruments used at each assessment.

#### Control variables

To make sure changes in the child's behavior are attributable to the intervention program, intervening life events that occur between the start of the program and during the follow up period, and treatment integrity are measured.

##### Intervening life events

To assess whether a change in the child's behavior is not attributable to stressful life events that occur between the start and finish of the program, in every session therapists keep score of new potential stressful or traumatic experiences in the life of the child. At T2 and T3, the post-test and follow-up assessments, parents fill out a checklist of life events in the last three (T2) or six (T3) months.

##### Treatment integrity

At least one, but preferably more parent and child sessions, of the intervention and control programs are randomly chosen and video-taped to be able to assess if the program is carried out according to the protocol. Ten percent of the video tapes will be randomly selected to be coded.

### Sample size calculation

The total sample size is based on the expected differences in outcome measures between children participating in the secondary prevention program 'En nu ik...!' and the control program 'Jij hoort erbij'. Because the effect of the intervention program has been tested before in a non-experimental design [22], a conservative estimate of f = 0,25 [59] is made about the effect size. Based on an alpha of .05, a power of .80 in a two tailed test, 48 subjects in each condition are needed.

To answer the research questions concerning the influence of potential mediators and moderators on the effectiveness of the secondary prevention program, several multiple regression analyses with different predictors will be performed. Based on an alpha of .05, an expected medium effect size of f^2 ^= 0.15 and a N = 90 we will be able to achieve a power of .80 for a model with up to five predictors. In smaller models of up to two predictors it will also be possible to draw conclusions about the independent contribution of each predictor.

Because of the tumultuous time these families are in we expect a relatively high drop-out rate of around 45% from T1 to T3. For this reason, we aim to recruit 250 children and parents.

### Drop-outs

All parents and children who consent to take part in the study will be followed from the moment of randomization till the follow-up assessment nine months later. To prevent drop-out parents and children are actively motivated by therapists and researchers to remain in the program and the study. Practical limitations are, as far as possible, removed, for example by arranging child care and transportation. If parents and children drop out of the program and are not able to come to the post-test and/or follow-up assessment, questionnaires will be mailed to the last known address and they are reminded by phone and email to complete the questionnaires. If children are not literate enough to fill out the questionnaires by themselves, only the parent is asked to fill out the questionnaire. The teacher is also sent a questionnaire by mail. If, after several reminders, the questionnaires are not sent back, the principle of last value carried forward will be applied. The reasons for drop-out will be documented and analyzed and the results will be reported back to the organizations participating in the study, so they may use this information to combat drop-out.

### Data analyses

Most analyses will be done using the software program SPSS. Intention-to-treat and completer analyses will be performed. Descriptive analyses will be carried out by standard methods. The change in behavior will be assessed on a continuous scale, taking into consideration the scale of the different variables. For analyses of the primary outcome measures, we plan to use ANOVA repeated measure analyses and the scores of the children in the intervention group will be compared with their own scores on a previous assessment, as well as with scores of children in the control group at the same assessment. Mediator and moderator models will be tested, using data of all children (intervention and control group). Analyses and reporting of the results will be done according to the CONSORT 2010 Statement-guidelines [60].

### Protection of data privacy

All parent-child dyads participating in the study will be assigned a number. Key lists will be stored separately from the data and will be deleted after final data analyses. Data will be analyzed in a way that no conclusions can be drawn about individual participants. All data are stored in lockable cabinets in lockable rooms. All employees and students working within the project sign a statement in which they declare not to disclose any information about research participants to a third party. Only if the safety of a parent or child is in danger, these concerns will be shared with the participating organization where the program is carried out.

### Publication policy

We plan to publish the results of this study in peer-reviewed, international journals. To make the results also available for Dutch policy makers and service providers, we plan to publish the results in (Dutch) journals within the field of youth mental health care. Results will be presented at international scientific conferences, as well as at national conferences within the field of youth care and mental health care.

### Ethical considerations

The study protocol is approved by the Medical Ethics Committee of the VU University Medical Center in Amsterdam, the Netherlands (METc VUmc 2009/99/NL26649.029.09). If changes to the study procedures are necessary they will be proposed to the Medical Ethics Committee as amendments. All changes will be described and discussed in the publication of the study's results.

## Discussion

Randomized studies into the effectiveness of preventive care for children exposed to interparental violence are scarce [6]. Part of the reasons may be that professionals are reluctant to deny or delay intervention for children at risk [23]. This obstacle was overcome by focusing on components of effectiveness and on mechanisms of change. This allows for the design of a control program in which children and parents receive an amount of attention that is acceptable to the professionals. This focus on components and mechanisms also provides a positive and constructive perspective on building and improving social responses to exposure to interparental violence, rather than highlighting a controlling stance towards the work of professionals. Insight into how the intervention works can help improve the program, if necessary, to maximize preventive effects and ensure that critical features are generalized to clinical practice. Insight into the question for whom preventive care works the best, can help develop criteria which will contribute to a more effective screening at the intake.

The use of multiple informants (parent, child and teacher) and independent observations will prevent reporting-bias. Multiple assessments are used to assess long-term consequences of the intervention and mechanisms of change. The converging of information will improve the reliability and validity of the collected information considerably [61]. 'En nu ik...!' is an easily accessible secondary prevention program for all children who experienced interparental violence and not just for a clinical subgroup or children residing in women shelters. A strength of this study is the recruitment of a representative broad subsample of all families who experienced interparental violence through different organizations across the entire country.

A specific problem with research in the context of marital conflict is that the conflict may extend to disagreement about children's participation in intervention and research. By Dutch law, both custodial parents have to be informed about and consent to participation of their child in a study. In consultation with the Medical Ethics Committee we agreed that strict application of this law for this study would effectively block research. In agreement we decided to balance those judicial concerns and concerns about the safety of the family, and take maximum steps to obtain informed consent from both custodial parents. However, if the other parent can not be reached or does not respond, the child can participate with informed consent of only one parent. If a parent does not want to inform the (ex)partner about the study though, the child can not participate in the study. This may cause drop-out of the most severe cases of interparental violence.

In conclusion, this study will gain knowledge about the effectiveness of a psycho-educational secondary prevention program for children exposed to interparental violence. Apart from questions concerning the effectiveness of this trauma-focused program, this study will also provide insight into the necessity of specific features in prevention programs for this population and selection criteria for participation.

### Status of the trial

The study started in November 2008. After being granted permission by the Medical Ethics Committee to start including participants, the first research participants were included in the fall of 2009. At the moment data collection is in progress. We expect the main results to be published at the end of 2013.

## Competing interests

The authors declare that they have no competing interests.

## Authors' contributions

FLW, CS and JCDS obtained funding for the study. All authors contributed to the design of the study and the development of the control program. MMO coordinates the recruitment of participants and data collection during the study. FLW, CS and JCDS supervise the progress of the study. MMO wrote the manuscript. All authors contributed to the further writing of the manuscript. All authors read and approved the final manuscript.

## Authors' information

MMO is a child psychologist and PhD-student at VU University and EMGO Institute for Health and Care Research in Amsterdam, the Netherlands, working on this study. JCDS is a researcher at the VU University and EMGO Institute for Health and Care Research in Amsterdam, the Netherlands. She has research experience in the field of child-caregiver relationships and quality of care in clinical and non-clinical (group) settings. FLW is a honorary professor at the VU University and EMGO Institute for Health and Care Research in Amsterdam, the Netherlands. Nationally and internationally, both her research program, 'child maltreatment: prevention and interventions', and her clinical work contribute to knowledge transfer in the field of domestic violence, child (sexual) abuse and neglect. She is founder and was director of the first Children's and Youth Trauma Center in the Netherlands. CS is a professor at the VU University and EMGO Institute for Health and Care Research in Amsterdam, the Netherlands. He is project leader of several research and development projects funded by NWO (fundamental research), ZonMW (health research), and charity funds. His research program 'Challenges to childrearing' contributes to the field of child protection, mental health and youth care, by focusing on diagnosis and treatment of disordered attachment, attachment to foster parents, and attachment in residential treatment.
